# Regulated Expression of *Chromobox Homolog 5* Revealed in Tumors of *Apc^Min^*^/+^ ROSA11 Gene Trap Mice

**DOI:** 10.1534/g3.112.002436

**Published:** 2012-05-01

**Authors:** Andrew T. Thliveris, Linda Clipson, Lacy L. Sommer, Barry A. Schoenike, Jason R. Hasenstein, Cassandra L. Schlamp, Caroline M. Alexander, Michael A. Newton, William F. Dove, James M. Amos-Landgraf

**Affiliations:** * Department of Ophthalmology and Visual Sciences; ‡ McArdle Laboratory for Cancer Research, Department of Oncology; § Department of Neuroscience; ** Department of Statistics; †† Department of Biostatistics and Medical Informatics, and; ‡‡ Laboratory of Genetics, University of Wisconsin, Madison, Wisconsin 53706; † Surgery Service, William S. Middleton Memorial Veterans Hospital, Madison, Wisconsin 53705

**Keywords:** WNT signaling, cancer progenitor, lacZ reporter, Cre drivers

## Abstract

The gene-trap lacZ reporter insertion, *ROSA11*, in the *Cbx5* mouse gene illuminates the regulatory complexity of this locus in *Apc^Min^*^/+^ mice. The insertion site of the *β-Geo* gene-trap element lies in the 24-kb intron proximal to the coding region of *Cbx5*. Transcript analysis indicates that two promoters for *Cbx5* flank this insertion site. Heterozygotes for the insertion express lacZ widely in fetal tissues but show limited expression in adult tissues. In the intestine, strong expression is limited to proliferative zones of crypts and tumors. Homozygotes for *ROSA11*, found at a lower than Mendelian frequency, express reduced levels of the coding region transcript in normal tissues, using a downstream promoter. Analysis via real-time polymerase chain reaction indicates that the upstream promoter is the dominant promoter in normal epithelium and tumors. Bioinformatic analysis of the *Cbx5* locus indicates that WNT and its target transcription factor MYC can establish a feedback loop that may play a role in regulating the self-renewal of the normal intestinal epithelium and its tumors.

A core strategy of current efforts to understand development and neoplasia is to document differential patterns of gene expression and deduce the genetic control over those patterns. Documentation of the pattern of gene expression takes several forms: transcriptome analyses of whole tissues reveal changes in transcript levels measured by hybridization to arrays or by the comparison of transcript copy numbers identified by deep sequencing in differentiating or neoplastic lineages. Alternatively, spatial and temporal patterns of gene expression can be visualized at cellular resolution by tagging each active gene using random transposon insertions. Here, a marker tag enables both the cloning of the active gene and visualization of its activity (Starr *et al.* 2011).

The genetic control of developmental and neoplastic processes is deduced by analysis of changes in spatial or temporal expression patterns that are elicited by mutations in relevant genes. These mutations may be studied singly or in combination. Loss-of-function alleles tagged with reporters provide a particularly powerful entry into the analysis of a system of genetic control. The heterozygote displays the pattern of expression of the gene of interest (Austin *et al.* 2004), and the homozygote reveals whether the gene’s activity is a necessary element in a biological feedback loop with positive or negative parity (Brenner *et al.* 1990; Thomas 1998).

Soriano *et al.* initiated the transposon-tagging approach to study development in the mouse by transforming embryonic stem (ES) cells with the promoter-trap vector ROSAβ-geo1-29. This reporter gene encodes a fusion protein, β-Geo, with both β-galactosidase (β-GAL) and neomycin phosphotransferase activity (Friedrich and Soriano 1991). One of the strains developed in this program, ROSA11 (R11), has drawn our attention because it expresses a β-GAL differentiation marker that is strongly expressed in the proliferative zone of intestinal crypts and in intestinal adenomas of Min mice (Gould and Dove 1996). By contrast, another of Soriano *et al.*’s promoter trap mouse strains, ROSA26, is expressed pervasively in the adult mouse and serves as a clonal lineage marker (Merritt *et al.* 1997; Zambrowicz *et al.* 1997; Thliveris *et al.* 2005).

This report provides evidence that the *R11* insertion lies within the heterochromatin protein 1α locus *Cbx5* on mouse chromosome 15. A detailed informatic analysis of the structure of this locus and a molecular analysis of its expression in normal and neoplastic tissues has uncovered a complex system of regulation of this locus. Our understanding of the biology of the normal self-renewing intestinal epithelium and its neoplastic derivative is enhanced by these observations.

## Materials and Methods

### Mouse strains, breeding, and maintenance

The congenic C57BL/6 (B6) R11 strain was derived from a single heterozygous male generously provided by P. Soriano (Baylor University, Houston TX) by backcrossing to B6 for 10 generations. The congenic B6 *Apc^Min/^*^+^ mice were obtained from our colony (Moser *et al.* 1990). The doubly heterozygous *R11/+ Min/+* animals were obtained by crossing *R11/+* females to *Min/+* males. Homozygous *R11/R11 Min/+* mice were obtained by crossing *R11/+* females to *R11/+ Min/+* males. Mice were maintained under a protocol approved by the Animal Care and Use Committee of the University of Wisconsin School of Medicine and Public Health and in a facility in the McArdle Laboratory approved by the American Association of Laboratory Animal Care. Animals were housed in standard caging with free access to mouse chow and acidified water.

### Histochemical staining for β-GAL activity

To understand the expression pattern of the *R11* promoter trap reporter, β-GAL activity was assayed in adult tissues. Mice were killed by CO_2_, and tissues were rapidly harvested, pinned on paraffin blocks, and fixed in freshly prepared 4% paraformaldehyde in phosphate-buffered saline (pH 7.3) on ice for 1 hr. Fixed samples were washed three times (30 min each) in Rinse Buffer [100 mM sodium phosphate (pH 7.0−7.5), 2 mM MgCl_2_, 0.01% sodium deoxycholate, and 0.02% Triton X-100]. Tissues were then stained for 12 to 14 hr in a humidified chamber at 37° in staining solution [Rinse Buffer plus 5 mM potassium ferricyanide, 5 mM potassium ferrocyanide, and 1 mg/mL X-GAL (Invitrogen, Carlsbad, CA) from a 25 mg/mL stock in dimethylformamide]. After staining, samples were rinsed in Rinse Buffer, post-fixed overnight in 10% formalin, and transferred to 70% ethanol. Tissues were embedded in paraffin and sectioned serially at 5 μm. Sections were counterstained with Nuclear Fast Red.

### Cloning of the *R11* insertion site using inverse PCR

Inverse polymerase chain reaction (PCR) was used to clone the genomic insertion site of the R11 promoter trap vector. The inverse PCR protocol was modified from that of Joslyn *et al.* (1991) as follows: total genomic DNA was isolated from spleens of B6 *R11/+* mice. A total of 16 μg of DNA was digested at 37° nearly to completion, first with *EcoR*I and then with *Hind*III. The restriction enzymes were then inactivated at 65° for 20 min. Digested DNA molecules were ligated (T4 DNA ligase #10799009001, Roche, at a concentration of 5−10 ng/μL) to maximize circularization (Ochman *et al.* 1990). Ligated DNAs were precipitated, washed, and resuspended in 40 μL of TE-4 [10 mM Tris (pH 7.5), 0.1 mM EDTA]. Five microliters of the ligated material was used for PCR experiments. Long-range PCR was performed by the Roche Diagnostics protocol (kit no. 11681834001) by using primers βGeo-D and SupF-A for the *Hind*III digest. (All primer sequences are provided in Table 1.) The PCR products were resolved on a 1% agarose gel and stained with ethidium bromide. A ~1700-bp band was visualized on an ultraviolet light box and excised. Agarose was removed from the gel fragment using kit no. 28704 from QIAGEN (Valencia, CA). The 1700-bp DNA fragment was then sequenced using a standard Sanger protocol (Applied Biosystems, Carlsbad, CA) with separate βGeo-D and SupF-A sequencing primers.

**Table 1:** Sequences of primers used for genomic PCR, RT-PCR, and real-time PCR analyses

Primer	Sequence
β-actin-F	5′ AACCCTAAGGCCAACCGTGAAAAG
β-actin-R	5′ TGGCGTGAGGGAGAGCATAGC
βGeo-D (a)	5′ TTGAGGACAAACTCTTCGCGGTC
SupF-A (b)	5′ GGCAGATTTAGAGTCTGCTCC
R11-G2L (c)	5′ CAGATCAGGCTTGACAGCAA
R11-G4 (d)	5′ GAGCAGCAAGCTCTACCAAG
GSP-1	5′ CCGTGCATCTGCCAGTTTGAGGGGA
GSP-2	5′ CGACGACAGTATCGGCCTCA
GSP-3	5′ CAGCTTTCCGGCACCGCTTC
Q_T_	5′ CCAGTGAGCAGAGTGACGAGGACTCGAGCTCAAGC(T)17
Q_0_	5′ CCAGTGAGCAGAGTGACG
Q_1_	5′ GAGGACTCGAGCTCAAGC
Ex1C-F (e)	5′ CGTGCAGGCCTTAGCGTGAGTGAT
E2/3R	5′ TATGGCCACCAGGTTCCGGAT
E2F4	5′ GATCTGCCAGCCCGCCAA
E3R3	5′ GGCTGTCCTCTTGGTCTTC
Hp1-BF (f)	5′ CAGGGAAGGATCCGTTTTG
Hp-1-1R (g)	5′ TGCACGGTTCCTCTGTGGTA
βGeoC-R (h)	5′ CAGCTGGCGAAAGGGGGATGTG
Hp1-2F (i)	5′ ATCTGCCAGCCCGCCAAG
Hp1-2R (j)	5′ ATCGCCCCAGTTCGTTCTTT
Hp1-4R (k)	5′ GCCTGTCCAACACCTTTTCC
Hp1-4F (l)	5′ GCACACGACATGGGAAAGAA
Hp1-5R (m)	5′ CAGCGCTGTTGGAGAAACTG
Insertion site of *R11*	5′ TTTTGTTTTT**AT**GTTAGTCTTATT

**Figure 1:**
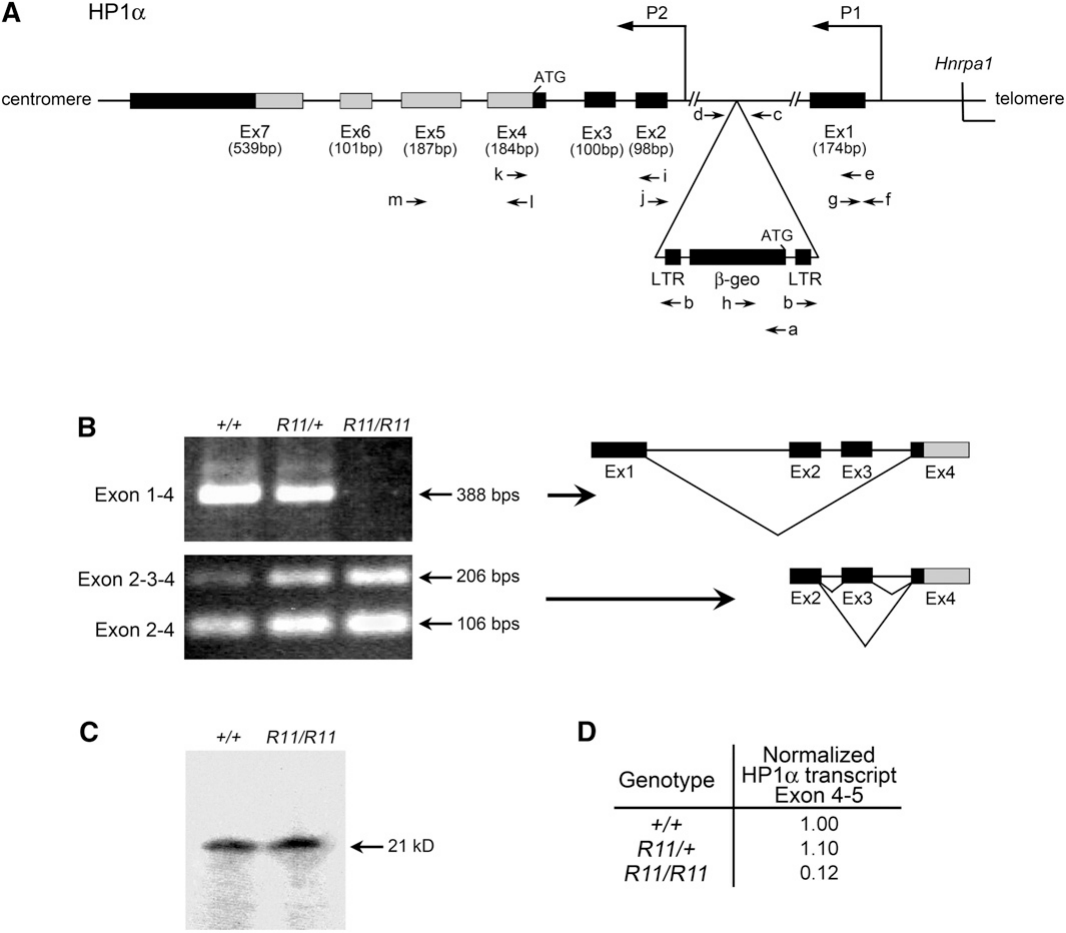
Structure and transcript profile of the of *Cbx5* region. (A) Intron−exon structure of the *Cbx5* locus located on mouse chromosome 15. Primers used (a−m) in genotyping, exon connection, and real-time PCR are listed in Table 1. 5′ untranslated exons (Ex1−3) and coding exons (Ex4−7) are designated. (B) Exon connection analysis using primers Hp1-BF and Hp1-4R in exons 1−4 and primers Hp1-2F and Hp1-BF in exons 2−4 using cDNA from spleens of +/+, *R11/+*, and *R11/R11* B6 animals. Products were resolved by agarose gel electrophoresis and stained with ethidium bromide. The splice form exon 1−4 was absent in the *R11/R11* homozygote. (C) Western blot analysis of total protein extracts from spleens, probed with a mouse monoclonal CBX5 antibody, as described in *Materials and Methods*. Intact CBX5 protein was present in both the wild-type and *R11/R11* homozygote. (D) Transcript levels in spleen RNA determined by real-time PCR, as described in *Materials and Methods*. A marked reduction in copy number of coding transcript (*Cbx5* exon 4−5) is noted in the *R11/R11* homozygote. This finding is consistent with the notion that the upstream promoter (P1) is responsible for the majority of the coding transcript of *Cbx5*.

Each letter in parentheses corresponds to the location of that primer relative to the genomic map of the *R11-Cbx5* locus in Figure 1. Bolded nucleotides represent the genomic insertion site of the *R11* promoter trap vector. PCR, polymerase chain reaction, RT, reverse transcription.

### Genotyping the *R11* and wild-type alleles in crosses

After the *R11* insertion site was determined, primer pairs (R11-G2L/R11-G4 and SupF-A/R11-G4) were synthesized to distinguish between the wild-type and the *R11* alleles, respectively. Progeny mice from crosses were genotyped from tail snip DNA using a three primer system with Primers R11-G2L/R11-G4/and SupF-A (Table1). Standard PCR conditions were used with 2.0 mM MgCl_2_ and 0.8 μM of each primer (final concentration). PCR was performed with 30 cycles under the following cycling conditions: 94° (30 sec), 60° (30 sec), and 72° (1 min). The PCR product was run on a 2% agarose gel for size fractionation. The product size of the *R11* fragment is ~500 bp, and the wild-type fragment is ~100 bp.

### Cloning by 5′ RACE-PCR of sequences downstream of the *R11* insertion

RACE-PCR was used to identify the promoter responsible for gene expression driven by the *R11* promoter trap insertion. Total RNA was isolated from the spleens of *R11/+* mice using TRIzol reagent (Invitrogen). A total of 80 μg of total RNA was used in the 5′ RACE protocol (Marathon cDNA Amplication Kit PT1115-1; Clontech, Mountain View, CA). Here, first-strand synthesis was performed using 1 μL of total RNA (7 μg/μL) added to 1 μL of GSP-1 primer and 10 μL of reagent-grade dH_2_O. This mixture was heated to 80° for 5 min and rapidly chilled on ice. A total of 8 μL of Reverse Transcription (RT) Mix [4 μL of 5X-RT Buffer (Invitrogen), 1 μL of 15 mM dNTP, 2 μL of 0.1 mM DDT, and 1 μL of Superscript II (Invitrogen)] were added and incubated at 42° for 90 min, 15 min at 50°, and 15 min at 70°. RNA was then digested by adding 1 μL of RNase H and incubating at 37° for 20 min. To tail the cDNA with dT, the entire cDNA mixture was added to a Centricon-50 column (Millipore, Billerica, MA), and the filtrate was concentrated *in vacuo* to a volume of 10 μL. Then, 10 μL of Tailing Mixture [4 μL of 5X Tailing Buffer (Invitrogen), 4 μL of 1 mM dATP, 1 μL of dH_2_O, and 1 μL of 10−20 U of TdT (Invitrogen)] were added to 10 μL of cDNA product and incubated at 37° for 10 min. Terminal transferase was then inactivated at 70° for 15 min.

#### 5′ RACE first round amplification:

A total of 1 μL each of a series of three test concentrations of dA tailed cDNA (direct, 1/25, 1/50) was added to 49 μL of First Round PCR Mixture [5 μL of 10X Gitschier’s Buffer (670 mM Tris, pH 8.8, 166 mM ammonium sulfate, 67 mM MgCL_2_), 5 μL of dNTP, 5 μL of dimethyl sulfoxide, 1 μL of 2μM Q_T_ primer, 1 μL of 25μM Q_0_ primer, and 1 μL of 25 μM GSP-2 primer, 31 μL of dH_2_O]. The reaction was cycled as follows: 98° (5 min), hold at 85° to add 1 μL of Taq (AmpliTaq; Perkin Elmer, Waltham, MA), then 29 cycles of 48° (2 min), 72° (40 min), 93° (1 min), 56° (1 min), and 72° (3 min), followed by 93° (1 min) and 72° (5 min) for product extension.

#### 5′ RACE second round amplification:

A total of 1 μL of a 1/20 dilution of the first round product was added to 49 μL of Second Round PCR Mixture (5 μL of 10X Gitschier’s buffer, 5 μL of dNTP, 5 μL of dimethyl sulfoxide, 1 μL of 25 μM Q_1_ primer, 1 μL of 25 μM GSP-3 primer, 1 μL of 1.25 U AmpliTaq, and 32 μL of dH_2_O). The reaction was cycled 39 times as follows: 98° (3 min), 93° (1 min), 56° (1 min), and 72° (3 min). The product was brought to 93° for 1 min followed by extension at 72° for 5 min. Then, 10 μL of this second PCR product was resolved on a 1% agarose gel, revealing a single ~400-bp band. This band was excised, purified, cloned with the TOPO-TA cloning kit (Invitrogen), and Sanger sequenced (using sequencing primer GSP-3) as described previously.

### Western blotting of CBX5 protein in *R11/R11 h*omozygotes

Western blot analysis as described by Schlamp and Nickells (Schlamp and Nickells 1996) was performed with the following modifications. Protein was isolated from B6 *R11/R11* spleens solubilized and sonicated in SBA [10% sodium dodecyl sulfate, 10 mM B-mercaptoethanol, 20% glycerol, 200 mM Tris (pH 6.8)]. Bradford analysis was used to determine the protein concentration in each sample. Each lane was loaded with 50 μg of soluble protein and the protein profile resolved on a 10% sodium dodecyl sulfate polyacrylamide gel, along with standard molecular weight markers. Gels were transblotted onto Immobilon P membranes (Millipore), visualized using Ponceau S, and blocked for 2 hr at room temperature in Tris-buffered saline (TBS) containing 5% skim milk powder. Blots were then washed extensively in TTBS (TBS plus 0.05% Tween 20) and incubated with primary antibody [10 μL of 1/200 dilution of mouse monoclonal to CBX5 protein (Ayyanathan *et al.* 2003) in 2 mL of Blocking Buffer (TBS and 5% skim milk)] overnight at 4° with continuous slow agitation. The blot was washed as above and challenged for 3 hr at room temperature with agitation using 1/2000 goat antirabbit secondary antibody conjugated to alkaline phosphatase (Jackson ImmunoResearch Laboratories Inc., West Grove, PA.) Polypeptides were visualized after color staining by 5-bromo-4-chloro-3-indolyl-phosphate and nitro-blue tetrazolium.

### RNA isolation and real-time PCR analysis

RNA was isolated from tissues frozen in liquid nitrogen using Trizol reagent and cleaned up using RNeasy Columns (QIAGEN) per the manufacturer’s protocol. RNA quantity and purity were assessed by spectrophotometric analysis. Up to 10 μg of RNA was treated using TURBO DNA-free (Ambion, Austin, TX) and inactivated per the manufacturer’s protocol. cDNA was synthesized from 1 μg of DNase-treated RNA using random hexamers and M-MLV reverse transcriptase in the presence of SuperAseIn (Ambion). Quantitative real-time PCR was conducted using an MJ Research Opticon 2 real-time PCR cycler and SYBR Premix Ex Taq (Takara Bio Inc., Shiga, Japan). The reactions contained 200 ng of cDNA and 200 nM final concentration of the primer pairs listed in Table 1. Several housekeeping control genes were tested, including *GAPDH*, *H2A.Z*, *TBP*, *PGK-1*, *mSdha*, *Alas-1*, and *β-actin*. *β-actin* was found to be consistent between tumor and normal intestine and was used to normalize all *Cbx5* primer sets for each RT-PCR run. The *β-actin* primers used are shown in Table 1. In addition, *β-actin* plasmid clones of each of the *Cbx5* primer sets were serially diluted and coordinately run with each primer set to determine the absolute copy number for each RT-PCR run. After correction for primer efficiency, the final copy numbers were determined from Ct values normalized to the *β-actin* reactions for each sample.

## Results

### Analysis of the *R11* genomic insertion site and fusion transcript identifies the *Cbx5* locus encoding the CBX5 (heterochromatin 1α) protein

Inverse PCR was used to identify the insertion site of the *R11* trap vector. DNA was isolated from the spleens of *R11*/+ mice and digested with *Hind*III. A 1700-bp genomic fragment was isolated and sequenced: a novel joint created by the insertion site of the *R11* promoter trap vector was identified (Table 1). This site was located between the two upstream untranslated *Cbx5* exons at position 103,049,787 bp (mouse genome assembly NCBI37/mm9; Figure 1A) on chromosome 15 (Kent *et al.* 2002; Fujita *et al.* 2011). Mapping crosses (summarized below) confirmed the position of the *R11* insertion to the region containing the *Cbx5* gene on chromosome 15.

Using spleen RNA from a *R11/+* mouse, a 380-bp fusion transcript was isolated by 5′-RACE (Frohman *et al.* 1988). This fragment was sequenced, and a 90-bp unique region was identified. A database search (http://blast.ncbi.nlm.nih.gov/) localized this sequence to the 5′ UTR of *Cbx5*. Real-time PCR of the wild-type allele connected this 5′ unique sequence to the first coding exon of *Cbx5*.

### Two promoters control the transcription of the *Cbx5* locus

To investigate the impact of the *R11* promoter trap insertion allele on the CBX5 transcript profile, exon connection experiments were developed, using RT PCR. Primers connecting the *Cbx5* coding exons 4 to 5 confirmed that RNA isolated from *R11/R11* homozygous, *R11*/+ heterozygous, and +/+ wild-type mice each contained the full coding sequence of *Cbx5* in exons 4 and 5. Interestingly, the RT PCR splice form connecting exon 1 (located 5′ to the *R11* insertion site) to exon 4 of the *Cbx5* coding sequence was absent in spleen RNA from the *R11* homozygote (Figure 1B). This result was confirmed by quantitative RT PCR assays using the same primers in exons 1 and 4 (Figure 1D). The reduced level of the exon 1−4 splice form can be explained by the strong transcription terminator present at the 3′ end of the promoter-trap sequence (Friedrich and Soriano 1991).

To investigate further the effect of the *R11* promoter trap insertion on CBX5 RNA expression, exon connection analyses were used to analyze products from the *Cbx5* coding region. Several distinct populations of exon connection products were detected. Exon 1 transcripts were spliced directly to exon 4 but were never associated with exon 2 sequence (Figure 1B). Exon 2, located downstream of the *R11* insertion site, was connected to the downstream *Cbx5* coding sequence in exon 4 through splicing with exon 3 and rarely with an alternative exon between 2 and 3 designated 2a (Figure 1B and data not shown). Taken together, two populations of CBX5 transcripts were confirmed; one initiated at upstream promoter (P1) that contains the exon 4−5 coding region except when it is efficiently prematurely terminated by the *R11* insertion, and one initiated at a downstream promoter (P2) that also contains the full coding region. These findings are consistent with the validated RefSeq sequences of three distinct full-length Cbx5 cDNAs from two distinct promoters (P1: RefSeq NM_001076789; P2: RefSeqs NM_007626.3, NM_001110216.1). Together, these observations support a two-promoter regulatory structure for the *Cbx5* locus—upstream P1 and downstream P2.

Primers connecting exon 4 to exon 5 confirmed that RNA isolated from homozygous, heterozygous, and wild-type mice always carried the full coding sequence of *Cbx5*, although at reduced levels in the homozygote (Figure 1D). Analysis of CBX5 protein from the spleen revealed that full-sized protein was expressed in the homozygote (Figure 1C). Thus, homozygosity for the *R11* insertion does not completely eliminate expression of CBX5 protein in the spleen.

### β-GAL staining of adult tissues in *R11/+* heterozygous adult mice

**Figure 2:**
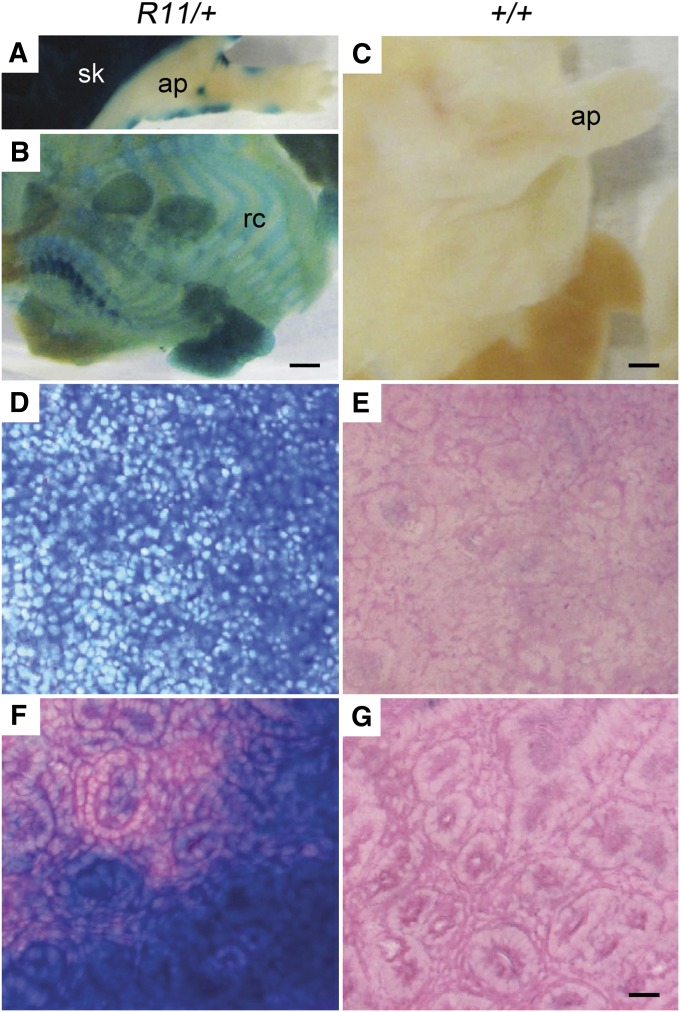
β-GAL expression in embryonic day 18 (E18) *R11/+* heterozygotes. (A) Whole *R11*/+ fetuses revealed ubiquitous staining of the skin adjacent to limited staining of the denuded E18 fetus. (B) By contrast, the denuded whole *R11*/+ fetus revealed β-GAL staining limited to the ribcage and appendage tissue types. (C) No staining was seen in the wild-type control E18 fetus. Ubiquitous staining was observed in *R11*/+ spleen (D) and kidney (F). No staining was noted in wildtype control spleen (E) and kidney (G). ap, appendage; rc, ribcage; sk, skin. Scale bar = 500 μm (A and B), 1000 μm (C), or 50 μm (D−G).

Widespread β-GAL staining in the *R11* heterozygous fetal and neonatal mouse was first described by Friedrich and Soriano (1991). In the current study, E18 heterozygotes exhibited extensive, but not uniform, staining in all tissues examined (Figure 2).

**Figure 3:**
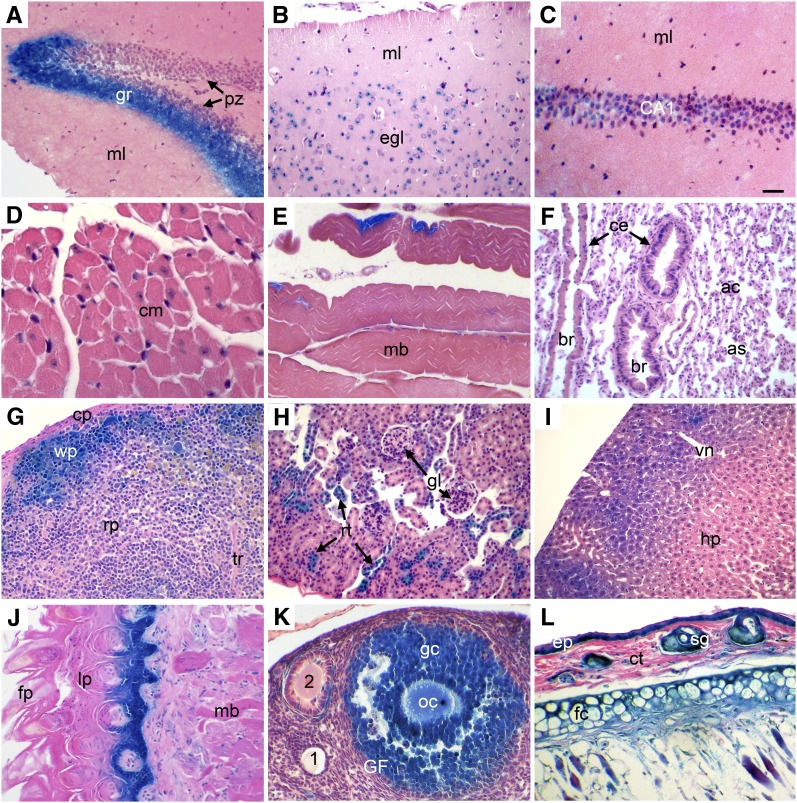
β-GAL expression in adult mouse tissues of *R11/+* heterozygotes. Adult tissues from *R11*/+ mice were stained with X-GAL to reveal β-GAL activity as described in *Materials and Methods*. (A, C) Hippocampus (gr, granular layer; ml, molecular layer; pz, proliferation zone; CA1, pyramidal cells). (B) Cerebral cortex (ml, molecular layer; egl, external granular layer). (D) Cardiac muscle (cm, cardiac muscle cell). (E) Skeletal muscle (mb, skeletal muscle bundle). (F) Lung (ac, alveolar cells; as, alveolar sac; br, bronchioles; ce, columnar cells). (G) Spleen (cp, capsule; rp, red pulp; tr, trabecula; wp, white pulp). (H) Kidney (rt, renal tubule; gl, glomerulus). (I) Liver (hp, hepatocytes; vn, vein). (J) Tongue (fp, filiform papilla; lp, lamina propria; mb, skeletal muscle bundles). (K) Ovary (1, primary follicle; 2, secondary follicle; gc, granulose cells; GF, Graafian follicle; oc, oocyte). (L) Skin (ct, connective tissue; ep, epidermis; fc, fat cells; gl: gland). All adult tissues stained positive for β-GAL except cardiac muscle (D) and lung (E). Scale bar = 100 μm (A, B, F−L) or 50 μm (C−E).

In the adult, the patterns of β-GAL expression were much more restricted, shown by the analysis of paraffin sections of various tissues from adult mice (Figure 3). Strong positive staining was localized to tissues presumed to be dividing, such as the lower proliferative zone in the small intestine and the germinal centers in the spleen. We also found β-GAL activity in nondividing tissues such as brain (Figure 3, A−C), skeletal muscle (Figure 3E), and the lung (Figure 3F). These patterns were found in both homozygous and heterozygous mice, whereas wild-type mice were negative.

### Effects of the *R11* insertion on viability assayed by transmission analysis

The family of Chromobox homolog proteins is involved not only in heterochromatic gene silencing but also in a broad range of functions encoded in euchromatin (reviewed by Kwon and Workman 2011). We therefore investigated whether the *R11* insertion element that blunts P1-driven expression from the locus affected the production of viable heterozygous and homozygous offspring. To enable a quantitative analysis of the effect of the *R11* insertion on transmission to viable B6 mice, genotyping primers were designed to distinguish among homozygous, heterozygous, and wild type. The transmission to viable progeny was investigated in two types of cross (Table 2). First, heterozygous mice were intercrossed. At 7 to 10 days, homozygous offspring were observed at a frequency of only 5%, rather than the expected 25%. Second, a similar deficiency in viable homozygous progeny was observed when heterozygous mice were backcrossed to surviving homozygotes. Thus, homozygotes survive at reduced frequencies, as scored at 7 to 10 days. Further, among the surviving adult *R11/R11* homozygotes, there were no overt phenotypic abnormalities. These survivors do not appear to have acquired linked genetic suppressors of lethality because they continue to transmit the *R11* insertion to homozygous progeny at reduced frequency.

**Table 2:** Transmission of the *R11* allele from *R11*/+ X *R11*/+ intercrosses and *R11*/+ X *R11*/*R11* backcrosses on the B6 background

	Cross			
	*R11*/+ X *R11*/+		*R11*/+ X *R11*/*R11*	
Genotype of Progeny	No. Observed	Expected	No. Observed	Expected
+/+	64 (38%)	25%	NA	NA
*R11*/+	95 (57%)	50%	95 (83%)	50%
*R11*/*R11*	8 (5%)	25%	20 (17%)	50%

Reported numbers represent live births tested at 7−10 days of age. The distribution of observed genotypes among the progeny from each cross differed significantly from the expected ratios for Mendelian segregation (*P* < 0.001, χ^2^ test). NA, not applicable.

### Induction of *Cbx5* in intestinal adenomas of *Apc^Min^*^/+^ mice

**Figure 4:**
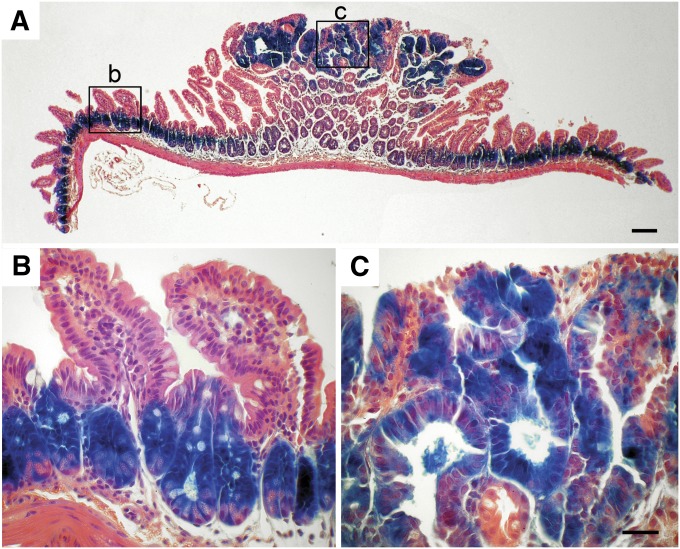
β-GAL expression in normal and adenomatous tissue in the small intestine. Tumor-bearing small intestine of a *R11* homozygous B6 *Apc^Min^*^/+^ mouse was stained with X-GAL to reveal β-GAL activity, as described in *Materials and Methods*. Lower (A) and higher (B-C) magnifications reveal β-GAL staining of the lower proliferative zone in the normal intestinal crypt (B) and the adenoma (C). Scale bar = 50 μm.

**Figure 5:**
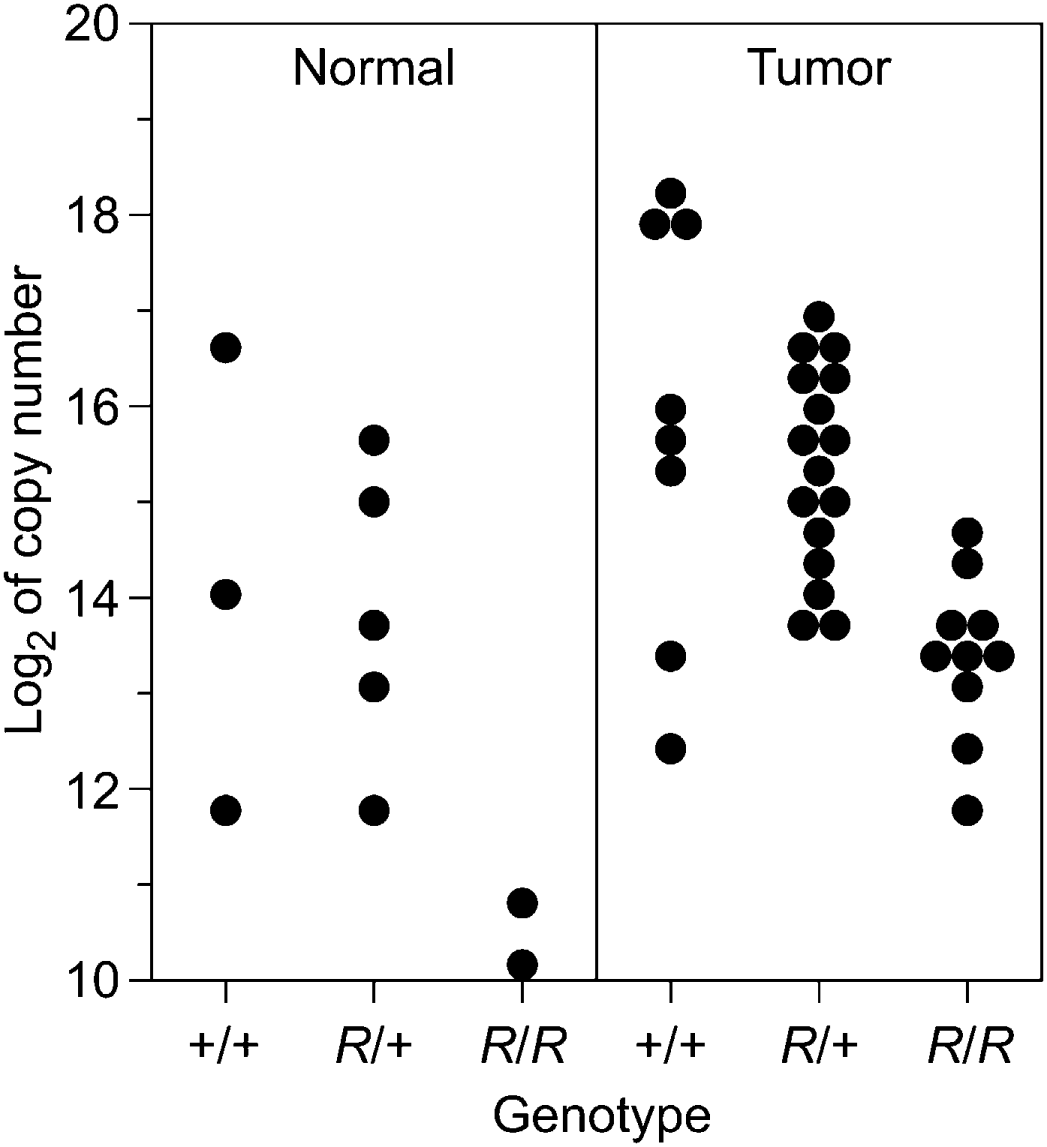
Expression level (base 2 logarithm of copy number) of *Cbx5*. Levels of the exon 4−5 coding region transcript in intestinal normal and tumor samples are shown for animals from three genotypic classes (+/+, *R11*/+, *R11*/*R11*). Two-way analysis of variance confirms a significant increase in average expression in tumors of 1.94 ± 0.55 (SE, log2 scale) compared with normal cells (*P* = 0.001) as well as a significant reduction in average expression in *R11/R11* homozygotes (−2.72 ± 0.64) as compared with wild type (*P* = 0.0001). The observed reduction in *R11* heterozygotes compared with wild type is not statistically significant (−0.77 ± 0.5, *P* = 0.18).

Strong β-GAL expression in the intestine of mice carrying the *R11* insertion is limited to the lower proliferative zone of crypts. Mice harboring the *Min* mutation of *Apc* in addition to the *R11* insertion give rise to β-GAL positive intestinal adenomas (Gould and Dove 1996, and Figure 4). Thus, the *lacZ* reporter element in the *R11* insertion serves as a developmental marker for the cells in the lower proliferative zone of the intestine and in *Min*-induced tumors. The level of CBX5 RNA expression within this developmental-neoplastic lineage was investigated by quantitative transcript analysis of normal and tumor tissue. CBX5 RNA transcripts harvested from equivalent amounts of tumor and normal intestinal tissue of B6 *Apc^Min^*^/+^ mice heterozygous and homozygous for the insertion were assayed by real time PCR (Figure 5). As a control, several housekeeping genes were assayed. β-actin was found to be the most consistently expressed across all tissues and was used to normalize RNA levels (see *Materials and Methods*). A two-way analysis of variance revealed a significant increase in average expression of CBX5 RNA in *R11/+* heterozygous tumor cells of 1.94 ± 0.55 SE (log2 scale) compared with normal cells (*P* = 0.001). In *R11/R11* homozygotes, the average expression of RNA from the exon 4−5 coding region of *Cbx5* was significantly reduced (−2.72 ± 0.64) as compared to wild type (*P* = 0.0001). In tumors, a nonsignificant reduction of CBX5 RNA level is observed in heterozygotes compared with wild type (−0.77 ± 0.5; *P* = 0.18). All *Min*-induced tumors differ from all normal tissue samples, and the three different genotypic classes differ from one another. However, within the restricted statistical power created by small numbers of certain classes in Figure 5, it cannot be concluded that these two sets of class differences are correlated.

## Discussion

The structure of the *R11* insertion has been established through the following series of steps. First, RACE PCR has shown that expression from the *β-Geo* promoter trap lies under the control of the *Cbx5* gene. A cDNA fusion product was identified that joined *β-Geo* sequence to an untranslated 5′-proximal exon of *Cbx5* (Figure 1). Next, inverse PCR analysis localized the *β-Geo* promoter trap vector to a 5′-proximal intron of *Cbx5* (Figure 1). Finally, exon connection experiments involving the 5′ exons of *Cbx5* gave products that are consistent with a model in which two distinct promoters (P1, upstream, and P2, downstream) regulate the transcription of this gene (Figure 1A). This model is consistent with the spliced expressed sequence tags identified in the GenBank genome database and visualized through the UC-Santa Cruz genome browser (http://genome.ucsc.edu/ and Kent 2002; Benson *et al.* 2004).

The sequence of the *Cbx5* locus suggests a number of interactions with transcription factors that reflect the *in vivo* expression pattern. The mouse *Cbx5* upstream promoter, P1, appears to be a bidirectional promoter shared with *hnRNPa1* that, by *in silico* analysis, has identifiable binding sites for *Myc* and *Tcf4* (Matys *et al.* 2006). Human Chip-Seq ENCODE data from multiple laboratories show direct binding of RNA polymerase 2, *My*c, *Tcf4*, and *c-Fos* at P1 (Rosenbloom *et al.* 2010). This is consistent with our observation of expression from the P1 promoter and expression of β-GAL activity in WNT-responsive *Min*-induced tumors (Figure 4). In these tumors *Tcf* and *β-catenin* are controlled by WNT signaling, leading to the expression of *Myc* (reviewed by Myant and Sansom 2011). The downstream promoter, P2, shows RNA polymerase 2 binding in human tumor and ES cell lines, supporting the observation that CBX5 function is not completely lost in the *R11* homozygous mouse (Figure 1, B and D). We hypothesize that, in the *R11/R11* homozygote, independent initiation of transcription at the P2 promoter drives exon 4−5 transcription during development that is suboptimal for viability (Table 2).

It is interesting to speculate on the role of the Cbx5 protein (heterochromatin 1α) in the biology of the crypt progenitor that is amplified in *Min*-induced tumors. This protein has been reported to interact with the origin recognition complex, ORF (Pak *et al.* 1997). High-resolution mapping analysis has shown that Cbx5 protein is also associated with transcriptionally active Drosophila chromatin (Cryderman *et al.* 2005). Cbx5 has been shown to be up-regulated in the breast cancer cell line MCF7 with corresponding increase in protein levels (Thomsen *et al.* 2011). The requirement for controlled replication of stem cells may involve an interaction between these classes of positive action by Cbx5 protein and an antagonist such as POF in Drosophila (Johansson *et al.* 2007).

Beyond the elements presumed to direct transcription, the *Cbx5* locus contains other conserved elements not likely to involve canonical promoters. Using the Multiz 30-way Alignment track in the UCSC genome browser (Blanchette *et al.* 2004, mouse genome assembly NCBI37/mm9, July 2007), five highly conserved noncoding regions of unknown function are identified in the first intron (24,380 kbp in total length). These conserved regions range between 500 and 1000 bp in length and are shared among mammals; four of the regions are found to be conserved further—through marsupials. The fifth region (between 103,057,150 and 103,057,450 on Chr. 15) shows a high level of conservation among mammals and extends to the chicken. No significant open reading frames can be found spanning these conserved regions. Using exon connection, we have observed no evidence for alternative splicing between exons 1 and 4 or for alternatively initiated transcripts upstream of P2. In addition, there is no evidence for a transcribed miRNA in the region on the basis of published miRNA database and prediction analysis (Griffiths-Jones 2004, 2006; Weber 2005; Griffiths-Jones *et al.* 2008). Despite the lack of evidence for transcription of these regions, their high level of conservation suggests that these are important noncoding regulatory elements. We shall return to discuss possible regulatory processes involving these conserved sequences in intron 1.

**Figure 6:**
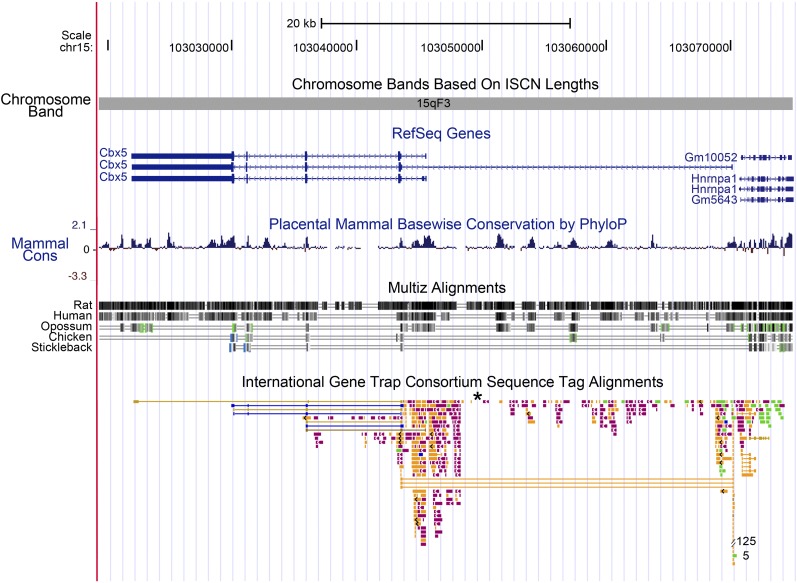
Physical map of mouse Chromosome 15 viewed centromere to telomere containing the *Cbx5* locus as visualized using the UCSC mouse genome browser (Kent *et al.* 2002; Fujita *et al.* 2011). The orientation of transcription is telomere to centromere with P1 oriented in the region 103,070,863 to 103,070,247, lying between *Hnrnpa1* and the first exon of *Cbx5*. P2 is oriented at 103,046,330 to 103,045,783. The tracks included are: basepair position, chromosome band, RefSeq genes (Pruitt *et al.* 2005) in blue, placental mammal basewise conservation by PhyloP (Siepel *et al.* 2005), Multiz alignments of human, rat, opossum, chicken, and stickleback (Blanchette *et al.* 2004), and the International Gene Trap Consortium sequence tag alignments (Skarnes *et al.* 2004), where we added an asterisk to indicate the position of the *R11* insertion. Peaks of mammalian conservation are recognized by PhyloP with high peak heights in the coding region of *Cbx5* and five peaks within intron 1. Two of these five intronic peaks show conservation between mammals and chicken, as shown by the Multiz alignment track. Gene trap sequence tags are color coded by the institution or source of the gene trap insertion and are available through the International Gene Trap Consortium (http://www.genetrap.org/).

Finally, the *Cbx5* region of the mouse genome is highly susceptible to transposon integration, deserving comment (Figure 6). The *R11* insertion lies near the second exon of *Cbx5*, within the conserved upstream promoter region. This region is an active area for transposon integrations. From among approximately 51,000 localized insertions that have been verified in the mouse genome, the International Gene Trap Consortium has identified and mapped 656 independent insertions within the *Cbx5* locus (To *et al.* 2004).This number represents a frequency of more than 1 per 1000 of the total reported insertions, nearly 20 times the frequency of insertions expected if the insertions into genes were random. Plausibly, this preference reflects the transcriptional activity of the *Cbx5* locus during development: active open chromatin is a preferred target for integration (Sandmeyer *et al.* 1990). Of the five highly conserved regions within intron 1 of the locus (Figure 6), insertions of transposons are found in or near the four conserved noncoding regions but not in the most highly conserved fifth region. Perhaps even in ES cells loss of a regulatory role of this element is lethal, or this element is refractory to integration because of its structure at the time of integration.

The function of the *Cbx5* locus has also been reflected by a histochemical analysis of β-GAL activity in fetal and adult tissues (Friedrich and Soriano 1991, and see *Materials and Methods*). In the E18 fetus, the *β-Geo* gene is expressed ubiquitously in the skin. The denuded E18 fetus reveals localized expression to appendages and rib cage. By contrast, ubiquitous expression of the β-GAL marker gene is found in the fetal spleen and kidney (Figure 2).

CBX5 function is particularly important for accurate mitotic division, as judged by siRNA down-regulation of the alpha, but not the beta and gamma isoforms in HeLa cells (De Koning *et al.* 2009). Current studies of chromatin structure associate CBX5 protein with late-replicating heterochromatin in mouse embryonic fibroblasts (Bachman *et al.* 2001). When somatic nuclei are reprogrammed with extracts from *Xenopus* oocytes, the level of CBX5 (heterochromatin 1α) protein in these nuclei decreases (Bian *et al.* 2009). Thus, we expect that the *Cbx5* gene has an essential function for viability and that this function is served by expression from both P1 and P2. Although the expression of P1 serves primarily in fetal tissues and dividing adult cells, it can also be observed in localized areas of even nonproliferating adult tissues tested including brain, cardiac muscle, and lung interstitium (Figure 3).

### Function in development and the two-promoter hypothesis

The *R11/R11* homozygote is not lethal but is found at frequencies reduced from those expected for Mendelian transmission: in heterozygous intercrosses, 5% of *R11/R11* mice survive to weaning instead of the predicted 25%. Among backcrosses of heterozygotes to homozygotes, there were 17% *R11/R11* mice instead of 50% (Table 2). The surviving homozygotes seem to be normal and fertile, and their progeny continue to display a low incidence of homozygous pups. Full-length CXB5 protein is expressed in the spleens of these *R11/R11* mice, as judged by Western analysis. However the predominant splice form for the *Cbx5* coding region, driven by the proposed upstream promoter, is absent in the *R11/R11 homozygote* (Figure 1, B and D). In the spleen, the level of exon 4−5 transcript from the coding region of *Cbx5* arises from a proposed promoter downstream of the *R11* insertion (Figure 1B) and is reduced by an order of magnitude in the *R11/R11* homozygote (Figure 1D; Figure 5).

### Function in neoplasms

In *Min*-induced adenomas of the small intestine, exon 4−5 transcripts of the *Cbx5* coding region are elevated compared to normal intestine. We interpret this elevation as a reflection of the amplification of the progenitor cell type in the tumor. As expected for this amplification model, this level of expression is observed in adenoma tissue for all three genotypes: +/+, *R11*/+, and *R11/R11*. The evidence for further deregulation of exon 4−5 transcript levels in *R11/R11* tumors compared to normal epithelium is not statistically significant (Figure 5; *P* = 0.61).

The expression of transcripts containing exon 4−5 in *Min*-induced adenomas deserves comment in light of the binding specificities inferred from the ENCODE Chip-Seq data (Rosenbloom *et al.* 2010). For the proposed upstream promoter, P1, both TCF and MYC binding are inferred and have been shown to be active in breast cancer cell lines (Thomsen *et al.* 2011). Because MYC is a direct downstream target of WNT-dependent TCF function (via β-catenin), this pair of binding activities formally constitutes a feedback loop. If MYC induces transcription from P1, the inferred feedback loop would have positive parity and contribute to the developmental choice in the normal crypt progenitor population and its amplified neoplastic derivative. By contrast, if MYC represses transcription from P1, the inferred feedback loop would have negative parity and would serve a homeostatic function in regulating WNT-induced CBX5 function. (The WNT-inhibitory function WIF1 may serve that same homeostatic regulatory logic.)

The observation that exon 4−5 transcript levels are also expressed in *Min*-induced tumors homozygous for the *R11* insertion is paradoxical in light of the aforementioned ENCODE analysis (Rosenbloom *et al.* 2010). How would these levels be driven if the distal P2 region shows no TCF or MYC binding specificity? One possible resolution to this issue would involve an additional transcription factor controlled by β-catenin, driving transcription from P2. Another conceivable resolution to this issue would involve long-range chromatin interaction with P2, either by P1 or by a locus control region elsewhere in the genome that is responsive to WNT and β-catenin.

The intricacies of gene expression uncovered by this initial analysis of the *R11* insertion in the *Cbx5* locus of the mouse genome have been developed by a molecular analysis of the spleen, the intestine, and *Min*-induced intestinal tumors, coupled with a bioinformatic analysis of the ENCODE database for the human genome. The observed expression of the locus in *Min*-induced adenomas is consistent with the bioinformatic prediction of WNT-induced transcription from the strong upstream promoter P1 and the known action of APC protein to regulate WNT signaling. Novel insight has come from the bioinformatic identification of a role for the WNT-dependent factor MYC also acting at promoter P1. The inferred feedback loop needs to be understood more fully in regard to its role in the homeostasis of the self-renewing normal intestinal epithelium and any subtle loss of control in the adenoma. Beyond these issues involving the strong promoter P1, the secondary promoter P2 raises the further issues of what drives its expression in normal tissues and in the adenoma (Figure 5).

Vermeulen *et al.* (2010) have observed that only a minority of cells in human colon cancers have activated WNT; these cells show stem cell activity in xenograft assays. We have argued that this class of cell is amplified in *Min*-induced adenomas. The promoters for the terminal developmental genes for fatty acid binding protein and villin, currently used to drive Cre recombinase in generating conditional gene knockouts, do not have this specificity for WNT and MYC. Thus, P1 (and perhaps P2) will be a useful new driver for experimentally manipulating the Min tumor lineage.
